# Functional Redundancy in Perchlorate and Nitrate Electron Transport Chains and Rewiring Respiratory Pathways to Alter Terminal Electron Acceptor Preference

**DOI:** 10.3389/fmicb.2018.00376

**Published:** 2018-03-06

**Authors:** Ouwei Wang, Ryan A. Melnyk, Misha G. Mehta-Kolte, Matthew D. Youngblut, Hans K. Carlson, John D. Coates

**Affiliations:** ^1^Department of Plant and Microbial Biology, University of California, Berkeley, Berkeley, CA, United States; ^2^Energy Biosciences Institute, University of California, Berkeley, Berkeley, CA, United States; ^3^Physical Biosciences Division, Lawrence Berkeley National Laboratory, Berkeley, CA, United States

**Keywords:** microbial perchlorate reduction, *Azospira suillum*, electron transport chain, microbial respiration, perchlorate, nitrate

## Abstract

Most dissimilatory perchlorate reducing bacteria (DPRB) are also capable of respiratory nitrate reduction, and preferentially utilize nitrate over perchlorate as a terminal electron acceptor. The similar domain architectures and phylogenetic relatedness of the nitrate and perchlorate respiratory complexes suggests a common evolutionary history and a potential for functionally redundant electron carriers. In this study, we identify key genetic redundancies in the electron transfer pathways from the quinone pool(s) to the terminal nitrate and perchlorate reductases in *Azospira suillum* PS (hereafter referred to as PS). We show that the putative quinol dehydrogenases, (PcrQ and NapC) and the soluble cytochrome electron carriers (PcrO and NapO) are functionally redundant under anaerobic growth conditions. We demonstrate that, when grown diauxically with both nitrate and perchlorate, the endogenous expression of NapC and NapO during the nitrate reduction phase was sufficient to completely erase any growth defect in the perchlorate reduction phase caused by deletion of *pcrQ* and/or *pcrO*. We leveraged our understanding of these genetic redundancies to make PS mutants with altered electron acceptor preferences. Deletion of the periplasmic nitrate reductase catalytic subunit, *napA*, led to preferential utilization of perchlorate even in the presence of equimolar nitrate, and deletion of the electron carrier proteins *napQ* and *napO*, resulted in concurrent reduction of nitrate and perchlorate. Our results demonstrate that nitrate and perchlorate respiratory pathways in PS share key functionally redundant electron transfer proteins and that mutagenesis of these proteins can be utilized as a strategy to alter the preferential usage of nitrate over perchlorate.

## Introduction

Perchlorate (${\text{ClO}}_{4}^{-}$) is deposited in the environment by both industrial activities and natural processes (Motzer, 2001; Urbansky, 2002; Dasgupta et al., 2005; Rajagopalan et al., 2009; Catling et al., 2010; Nilsson et al., 2013). Due to the lack of regulations for perchlorate disposal before 1997 and the high water solubility, perchlorate contamination is widespread and has been detected in a diversity of drinking water supplies (Urbansky, 2002; Rajagopalan et al., 2009). Perchlorate-contaminated water poses a potential health risk as it accumulates in food sources, inhibiting the uptake of iodine by the thyroid gland (Stanbury and Wyngaarden, 1952). This can disrupt thyroid hormone production leading to hypothyroidism, physical, and neuropsychological development anomalies (Stanbury and Wyngaarden, 1952; Wolff, 1998; Greer et al., 2002; Taylor et al., 2014).

Most approaches to long-term robust perchlorate remediation rely on microbial bioattenuation either *in situ* or *ex situ* through the use of bioreactors. Despite its human toxicity, perchlorate can be used as a terminal electron acceptor by dissimilatory perchlorate-reducing microorganisms (DPRM) during anaerobic respiration (Coates et al., 1999; Coates and Achenbach, 2004; Youngblut et al., 2016b; Wang and Coates, 2017). DPRM are phylogenetically diverse and can be isolated from many environments (Coates et al., 1999; Carlström et al., 2015). All isolated Gram-negative DPRM are facultative anaerobes, and the majority are capable of alternatively using nitrate as an electron acceptor (Herman and Frankenberger, 1999; Achenbach et al., 2001; Coates and Achenbach, 2004; Carlström et al., 2013, 2015). When both perchlorate and nitrate are present, pure, or mixed culture DPRM either preferentially or concurrently reduce nitrate, even though perchlorate respiration is energetically more favorable (*E*°′ = +797 mV for the reduction of ${\text{ClO}}_{4}^{-}$ to Cl^−^, *E*°′ = +750 mV for the reduction of ${\text{NO}}_{3}^{-}$ to N_2_) (Chaudhuri et al., 2002; Coates and Achenbach, 2004; Xiao and Roberts, 2013). This occurs regardless of whether the culture was previously grown on oxygen, nitrate, or perchlorate (Chaudhuri et al., 2002; Coates and Achenbach, 2004). The only known exception is *Sedimenticola selenatireducens* CUZ, in which, perchlorate is preferentially utilized if the inoculum is pre-grown on perchlorate (Carlström et al., 2015). Preferential utilization of nitrate by DPRM is non-intuitive and presents an obstacle to the facile bioremediation of perchlorate, as electron donor additions preferentially drive denitrification rather than perchlorate reduction in a bioreactor (Nozawa-Inoue et al., 2005; Choi and Silverstein, 2008). In most contaminated environments, nitrate concentrations can dominate perchlorate concentrations by several orders of magnitude. As such, attenuating perchlorate to regulatory standards often necessitates that the majority (>90%) of the applied electron donor is used to consume the nitrate. Consequently, new strategies to influence this electron acceptor utilization preference could improve treatment efficiency especially in environments where nitrate concentrations dominate (Youngblut et al., 2016b).

**Figure 1:**
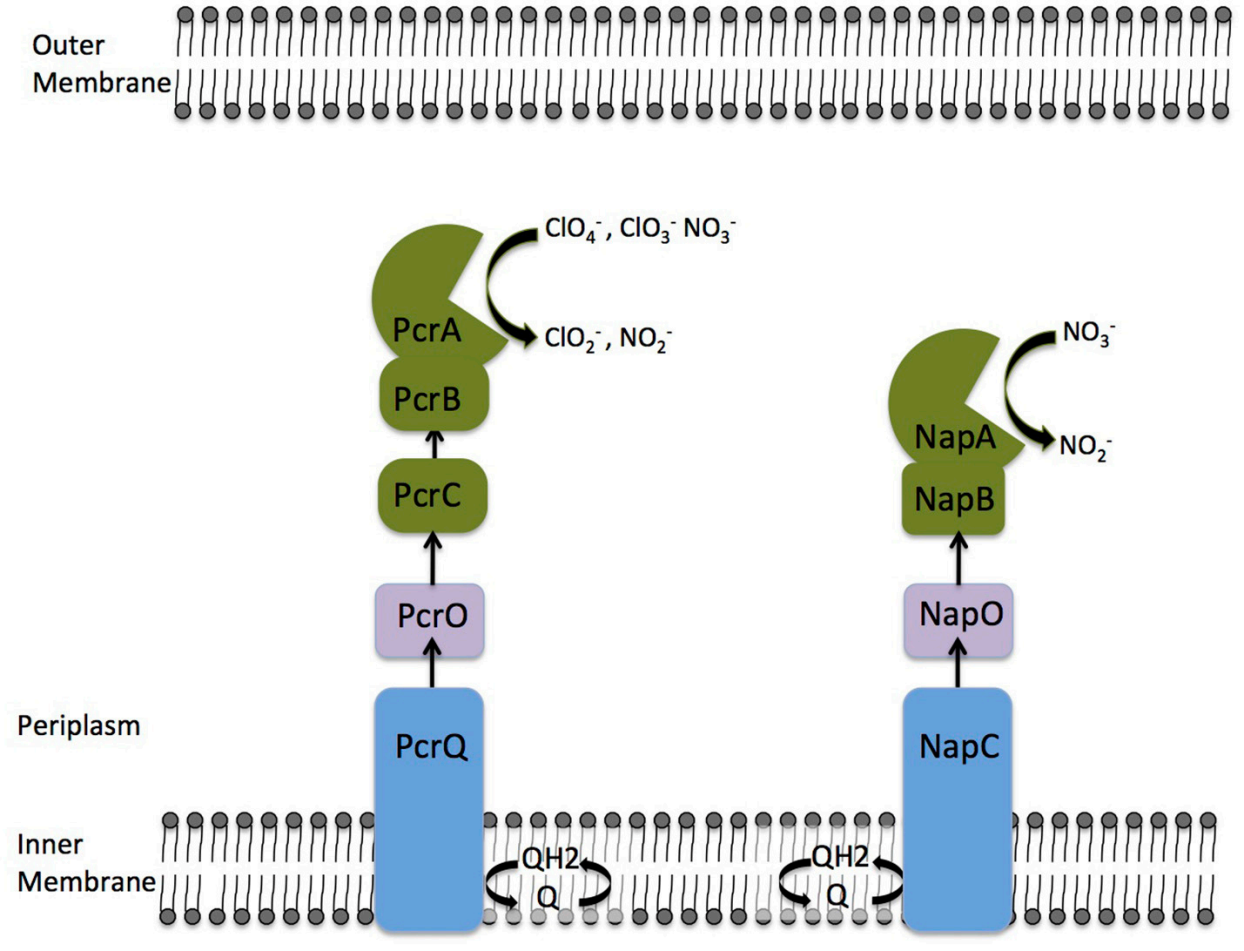
A model displaying proposed electron transfer networks between the quinone pool and PcrABC/NapAB respiratory complexes in *Azospira suillum* PS. Arrows indicate the canonical electron transfer pathways. The nitrate and perchlorate reductase complexes are painted green. Redundant electron transfer proteins are painted with the same color.

Previous comparative analysis of the genomes of DPRM identified a horizontally transferred perchlorate reduction genomic island (PRI) associated with perchlorate metabolism (Melnyk et al., 2011). Genomic and genetic analysis of PS perchlorate reduction machinery identified a PRI containing 17 genes and defined which genes are essential for respiratory perchlorate reduction (Melnyk et al., 2014). The genes *pcrABC* code for subunits of the perchlorate reductase complex (Melnyk et al., 2014; Youngblut et al., 2016a). From biochemical and genomic evidence, it is clear that reduced tetraheme *c*-type cytochrome PcrC donates electrons, via the iron-sulfur cluster protein PcrB, to the perchlorate reductase catalytic subunit PcrA, which reduces perchlorate to chlorite (${\text{ClO}}_{2}^{-}$) as the initial step in the pathway (Melnyk et al., 2014; Youngblut et al., 2016a,b; Mehta-Kolte et al., 2017). However, the precise electron transfer pathway from the quinone pool to PcrC has remained obscure. In the PRI, two proteins annotated as having redox active cofactors are likely candidates for electron carriers during perchlorate reduction. PcrO (*dsui_0143, pcrO)*, a diheme *c*-type cytochrome, and PcrQ (*dsui_0143, pcrQ)*, a tetraheme c-type cytochrome that likely functions as a quinol dehydrogenase (Melnyk et al., 2014). Consistent with a role in electron transfer during perchlorate respiration, Δ*pcrQ* and Δ*pcrO* strains displayed slower growth kinetics with perchlorate as an electron acceptor, however, remarkably, neither gene was essential for perchlorate reduction (Melnyk et al., 2014). This observation led to the central hypothesis and motivation for this study that functionally redundant electron transport proteins, likely from the nitrate respiratory pathway, are capable of complementing for the function of *pcrQ* and *pcrO* (Figure 1).

## Materials and methods

### Bacterial strain cultivation and characterization

Wild type *Azospira suillum* PS (Michaelidou et al., 2000) (Isolated by the Coates lab, ATCC^R^ BAA-33, hereafter referred to as wtPS) and mutant strains were revived from freezer stocks by streaking out for single colonies on ALP medium agar plates (Melnyk et al., 2014). One liter of ALP medium is composed of 0.49 g monobasic sodium phosphate dihydrate, 0.97 g dibasic anhydrous sodium phosphate, 0.1 g potassium chloride, 0.25 g ammonium chloride, 0.82 g sodium acetate, 2.0 g yeast extract, 7.6 g of a 60% (wt/wt) sodium lactate solution, 1.10 g sodium pyruvate, and 10 ml of both vitamin mix and mineral mix as previously described (Bruce et al., 1999). To make solid ALP medium for plates, 15 g/L agar was added. For aerobic cultivation, single colonies from plates were picked into LMM medium and cultivated at 37°C with vigorous shaking (250 rpm). LMM minimal medium is composed of the same ingredients as ALP, but omitting acetate, pyruvate, and yeast extract. For anaerobic cultivation, liquid overnight aerobic cultures were used to inoculate anoxic LMM medium amended with 5 mM each of sodium nitrate and sodium perchlorate. Media were made anoxic by flushing with oxygen-free N_2_, and sealed with butyl rubber stoppers prior to autoclaving. Anaerobic cultures were cultivated in anaerobic tubes (Bellco) with a nitrogen headspace at 37°C without shaking.

All phenotypic analyses to compare strains were carried out in microplate cultures. For microplate growth experiments, 2.5 μl of overnight stationary phase PS culture was used to inoculate 250 μl of LMM medium supplemented with nitrate (5 mM), perchlorate (2.5 mM), or both nitrate and perchlorate (5 mM each). After inoculation, 80 μl of mineral oil was added to each sample to prevent evaporation. The concentration of perchlorate under the perchlorate-only condition was lowered to avoid unpredictably long lag periods seen with high perchlorate concentrations (unpublished data), possibly due to enzymatic substrate inhibition observed previously for the PcrAB (Youngblut et al., 2016a). Strain characterization experiments were conducted in flat-bottom 96-well plates (Corning Costar, Tewksbury, MA) in an anaerobic chamber filled with 98% N_2_ and 2% H_2_ (Coy Labs, Grass Lake, MI). Note PS does not use hydrogen as an electron donor for energy metabolism (Michaelidou et al., 2000; Thrash et al., 2007). The optical density at 600 nm was measured with a spectrophotometer (Tecan Sunrise, Männedorf, Switzerland) at 37°C without shaking. All growth data were plotted and calculated with the Prism 7 software.

To test the preferential utilization of nitrate and perchlorate, 0.5 mL of overnight aerobic grown cells were transferred to anaerobic tubes containing 10 mL LMM medium with 5 mM each of sodium perchlorate and sodium nitrate. Cell growth was measured spectrophotometrically at 600 nm. All experimental analyses were performed in three biological replicates.

### Strain construction

In-frame gene deletions and complementation experiments in PS were conducted as previously described (Melnyk et al., 2014). Briefly, the suicide vector pNPTS138 (Dickon Alley via Kathleen Ryan, UC, Berkeley) with a gene deletion cassette was transformed into PS by electroporation using a method previously described for *Zymomonas mobilis* (Lam et al., 1993). Transformed PS cells were plated on ALP agar plates with 50 μg kanamycin/mL medium (ALP-KAN) to select for integration of the suicide vector into the chromosome. Resulting PS colonies were picked into ALP liquid medium, cultivated overnight, and plated on ALP-KAN and ALP-sucrose (6%) agar plates to select for colonies that were sensitive to kanamycin and resistant to sucrose. Colony PCR was used to screen for crossover mutants containing the gene deletion. The pBBR1MCS2 plasmid was used as the complementation vector to express *pcrQ, pcrO, napC, napO in trans* (Kovach et al., 1995). The PCR-amplified *pcrQ, pcrO, napC, napO* genes with their native ribosomal binding sites (defined as 20 nucleotides upstream of the start codon) were cloned downstream of the *nap* or *pcr* native promoters to generate complementation vectors. A 20–500 nucleotides region upstream of the first gene of the operon was treated as the native promoter (Melnyk et al., 2014). Complementation vectors were transformed into PS by electroporation as previously described (Melnyk et al., 2014). Strains, plasmids, and primers used for this study are listed in Tables S1–S3 respectively.

### Analytical procedures

Perchlorate and nitrate concentrations were measured via ion chromatography using a ICS 1500 (Dionex) equipped with an Ion Pac AS25 column (4 × 250 mm, Thermo Scientific) with a mobile phase of 36 mM NaOH at a flow rate of1.0 mL/min. Analyses were detected by conductivity suppressed with an ASRS 300 (4 mm, Dionex) in recycle mode. The suppressor controller was set at 90 mA for analysis. The injection volume was 10 μL. Cultures were sampled anaerobically using sterile 1 ml syringes, filtered with 0.2 μm syringe filters and diluted in deionized water.

## Results and discussion

### Addition of both nitrate and perchlorate rescues single deletion mutants in either quinol dehydrogenases (Δ*pcrQ*, Δ*napC*) or electron transfer cytochromes (Δ*pcrO*, Δ*napO*)

The *pcrQ* and *pcrO* genes are highly conserved among phylogenetically diverse DPRB, suggesting that, as in PS, they are important for perchlorate metabolism. However, the observation that *pcrQ* and *pcrO* are not absolutely essential for perchlorate reduction in PS suggests than their role can be partially complemented by other functionally redundant proteins in the PS genome (Melnyk et al., 2011; Melnyk and Coates, 2015). PS can also effectively utilize nitrate as an alternative terminal electron acceptor wherein the nitrate is reduced to nitrogen gas. BLAST-P searches with protein sequences against the PS genome (taxid:640081) identified two proteins (NapC and NapO) from the periplasmic nitrate reductase *nap* operon that are homologous to PcrQ and PcrO. PcrQ is 79% identical to the PS NapC homolog (locus tag: Dsui_1176) (Potter and Cole, 1999). Both PcrQ and NapC are tetraheme *c*-type cytochromes belonging to the NapC/NirT family (Potter and Cole, 1999), and NapC is known to function as a quinol dehydrogenase that mediates electron transfer between quinone pool and soluble periplasmic nitrate reductase (Roldán et al., 1998; Potter and Cole, 1999; Brondijk et al., 2002). PcrO is 55% identical to NapO (Dsui_1177), and like PcrO, NapO is also a diheme *c*-type cytochrome. Both NapO and PcrO are homologous to the structurally characterized gamma subunit in the ethylbenzene dehydrogenase complex from *Aromatoleum aromaticum* (Kloer et al., 2006). Of note, to our knowledge, no previous studies have identified *napO* as a part of denitrification pathway (Brondijk et al., 2002; Stewart et al., 2002; Kern et al., 2007; Simpson et al., 2010; Kraft et al., 2011; Sparacino-Watkins et al., 2014).

**Figure 2:**
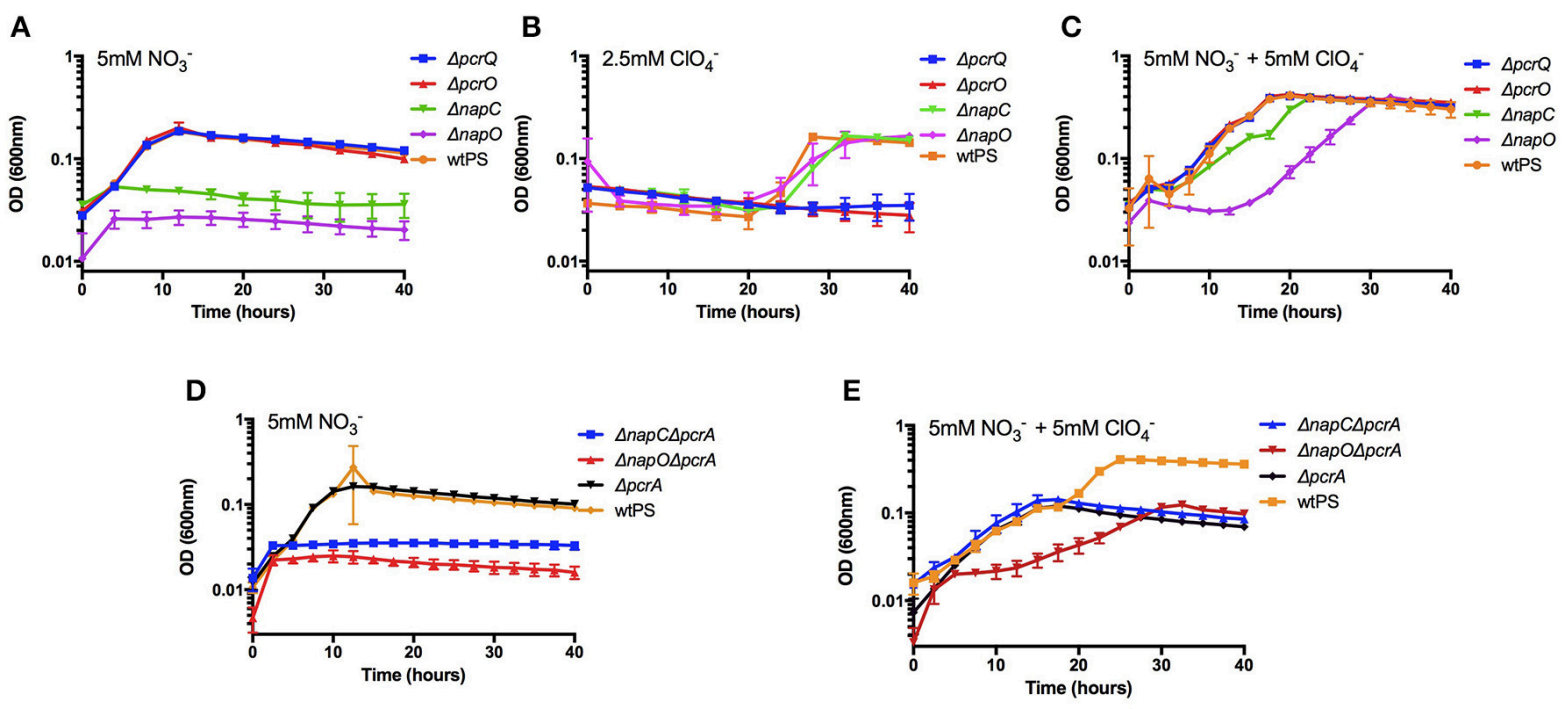
Growth of wtPS, Δ*pcrQ*, Δ*napC*, Δ*pcrO*, and Δ*napO* in LMM medium containing nitrate **(A)**, perchlorate **(B)**, both nitrate and perchlorate **(C)**. Growth of wtPS, Δ*pcrA*, Δ*napC*Δ*pcrA*, and Δ*pcrO*Δ*pcrA* in LMM medium containing nitrate **(D)**, and both nitrate and perchlorate **(E)**. Error bars represent standard deviations of biological triplicate samples.

It was previously observed that under nitrate reducing conditions, *pcrQ* and *pcrO* single deletion mutants (Δ*pcrQ*, Δ*pcrO*) had no growth defect and were in fact similar to wtPS (Melnyk et al., 2014). When grown with perchlorate, Δ*pcrQ* and Δ*pcrO* mutants displayed slightly slower growth kinetics but reach the same final optical density as wtPS (Melnyk et al., 2014). When grown diauxically with both nitrate and perchlorate, Δ*pcrQ* and Δ*pcrO* displayed no growth defect compared with wtPS (Melnyk et al., 2014). These results suggested that other genes in the PS genome are functionally redundant with *pcrQ* and *pcrO*. We hypothesized that rescue of Δ*pcrQ* and Δ*pcrO* growth defects in diauxic growth conditions is due to functional redundancy with *napC* and *napO* from the nitrate reduction pathway, which is induced by the presence of nitrate. Similarly, *napC* and *napO* would also not be essential for nitrate reduction because by corollary their electron transfer role should be rescued by *pcrQ* and *pcrO*. To demonstrate functional redundancies between the putative quinol dehydrogenases and the electron transfer cytochromes, *napC* and *napO* deletion mutants were constructed (Δ*napC*, Δ*napO*) and the growth phenotypes under nitrate, perchlorate, and diauxic growth conditions were tested. Δ*napC* and Δ*napO* mutants failed to grow when nitrate was the sole electron acceptor (Figure 2A). Under perchlorate growth conditions, both the Δ*napC* and Δ*napO* showed no growth defect compared to wtPS (Figure 2B). Interestingly, Δ*pcrQ* and Δ*pcrO* showed a more severe growth defect compared to the previous study (Melnyk et al., 2014), likely due to the LMM minimal medium used in this study lacks yeast extract, which could contain trace amount of nitrate to partially induce the expression of nitrate reduction pathway. However, under diauxic growth conditions, all mutants reached the same final optical density as wtPS (Figure 2C). Δ*napC* and Δ*napO* grew with an increased lag phase, and slightly slower growth rate comparing to wtPS over both phases of the diauxic growth assay (Figure 2C, Table S4). Δ*pcrQ* and Δ*pcrO* displayed no growth defect compared to wtPS, consistent with previous study (Melnyk et al., 2014). Taken together, these results support the hypothesis that the homologous putative quinol dehydrogenases (PcrQ and NapC) and soluble periplasmic electron transfer proteins (PcrO and NapO) are functionally redundant, and independently capable of rescuing growth defects due to the absence of their homolog counterparts. A summary of the growth rates of these strains can be found in Table S4.

### Δ*napC* and Δ*napO* growth over both phases of diauxic growth curves is not due to nitrate reduction by PcrABC

Biochemical results show that the perchlorate reductase enzyme complex, PcrABC, can reduce several substrates other than perchlorate, including nitrate (Kengen et al., 1999; Heinnickel et al., 2011; Youngblut et al., 2016a). Thus, one explanation for the rescue of the Δ*napC* and Δ*napO* growth defects under the diauxic growth condition is that expression of the *pcr* genes leads to production of a fully functional perchlorate and nitrate reductase that facilitates respiratory nitrate and perchlorate reduction. To address this possibility, we deleted *pcrA* in Δ*napC* and Δ*napO* single mutants to construct Δ*napC*Δ*pcrA and* Δ*napO*Δ*pcrA* double mutants. With nitrate as the sole electron acceptor, Δ*napC*Δ*pcrA* and Δ*napO*Δ*pcrA* strains displayed the identical growth defect as Δ*napC* and Δ*napO* (Figures 2A,D). As expected, Δ*napC*Δ*pcrA*, Δ*napO*Δ*pcrA, and* Δ*pcrA* were not viable when perchlorate was the sole electron acceptor. When grown diauxically with both nitrate and perchlorate, all strains with *pcrA* deletion are incapable of growing over the second perchlorate growth phase (Figure 2E). Δ*napC*Δ*pcrA* grew over the nitrate phase of the diauxic growth curve with a similar rate to wtPS and Δ*pcrA*, while Δ*napO*Δ*pcrA* grew with a prolonged lag phase and a slightly slower growth rate (Table S4). Both double mutants Δ*napC*Δ*pcrA* and Δ*napO*Δ*pcrA* reached the same final optical density as Δ*pcrA* in the diauxic growth condition (Figure 2E). These results confirm that Δ*napC* and Δ*napO* electron transfer functions to the nitrate reductase complex are rescued by perchlorate induced electron carriers and that their phenotypes are not due to nitrate reduction by PcrABC.

There are two *nap* operons in PS, *napDAGHB* and *napCOF*, which are located in different regions in the genome. Based on previous work with *Escherichia coli* K-12, NapG and NapH form an alternate non-essential quinol dehydrogenase that transfer electrons to NapAB, via NapC (Brondijk et al., 2002). While deletion of *napGH* in *E. coli* resulted in a mild growth defect (Brondijk et al., 2002), deletion of *napGH* in PS did not result in any significant growth defect under all conditions tested (Figure S1). This indicates that under the tested conditions NapGH is not donating electrons to either PcrQ or NapC. Further investigations are needed to define the exact function of NapGH in PS.

### Functional redundancy of *napC*/*pcrQ* and, *napO*/*pcrO*

**Figure 3:**
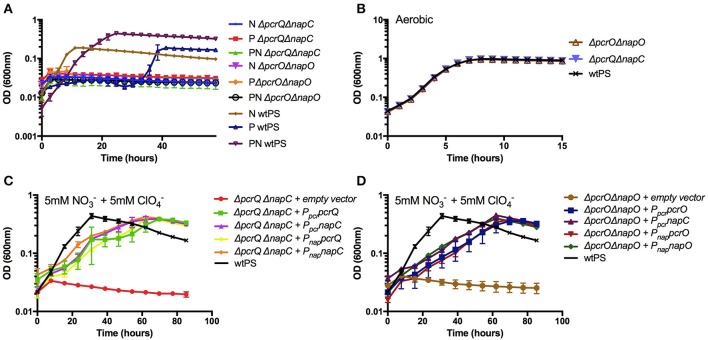
Growth of Δ*pcrQ*Δ*napC* and Δ*pcrO*Δ*napO*. **(A)** Growth phenotype of wtPS, Δ*pcrQ*Δ*napC*, and Δ*pcrO*Δ*napO*, on LMM medium containing nitrate (N), perchlorate (P), both nitrate and perchlorate (PN), and **(B)** oxygen were tested. **(C,D)** Growth of Δ*pcrQ*Δ*napC* with complementation vectors expressing *pcrQ or napC*, and Δ*pcrO*Δ*napO* with complementation vectors expressing *pcrO or napO*, on LMM medium with nitrate and perchlorate. Complementation tests were conducted with either *pcr* or *nap* promoter (P_*pcr*_ and P_*nap*_). Error bars represent standard deviations of biological triplicate samples.

While NapC is homologous with PcrQ and NapO is homologous with PcrO, and our results clearly show cross-complementation of mutant growth defects by some component of the nitrate or perchlorate regulon, we sought to conclusively demonstrate the functional redundancy of the homologs PcrQ/NapC and PcrO/NapO. We constructed Δ*pcrQ*Δ*napC* and Δ*pcrO*Δ*napO* and confirmed that, while capable of aerobic growth, these strains were incapable of anaerobic growth in any of nitrate, perchlorate, or diauxic growth conditions (Figures 3A,B, Table S5).

To further confirm the functional redundancy between *napC* and *pcrQ*, and between *napO* and *pcrO*, we attempted to complement the double deletion mutants Δ*pcrQ*Δ*napC* and Δ*pcrO*Δ*napO* by introducing *pcrQ, napC*, and *pcrO, napO in trans*, under either the *pcr* or *nap* promoter. Δ*pcrQ*Δ*napC* and Δ*pcrO*Δ*napO* harboring different complementation plasmids were subjected to growth experiments under nitrate and perchlorate diauxic growth conditions (Figures 3C,D). Consistent with a functional redundancy, Δ*pcrQ*Δ*napC* on nitrate or perchlorate was complemented by either *pcrQ* or *napC*, and Δ*pcrO*Δ*napO* was complemented by either *pcrO* or *napO* (Figures 3C,D). The complementation was achieved under either the *pcr* or *nap* promoter. All complemented strains reached the same optical density as wtPS but displayed defective growth kinetics, which was likely due to the disruption of NapC, NapO, PcrQ, or PcrO protein expression level by increased gene copy number *in trans* and/or regulatory issues associated with the use of native *nap* or *pcr* promoters. Taken together, these results show that the components of the electron transport conduits from the quinone pool to NapAB or PcrABC, are functionally redundant. No other electron transfer protein expressed in PS under our growth conditions could function as quinol dehydrogenase or intermediate electron carrier to the nitrate or perchlorate reductase complexes.

### Four redundant electron transfer routes are functional from the quinone pool to either terminal reductases—PcrABC or NapAB

**Figure 4:**
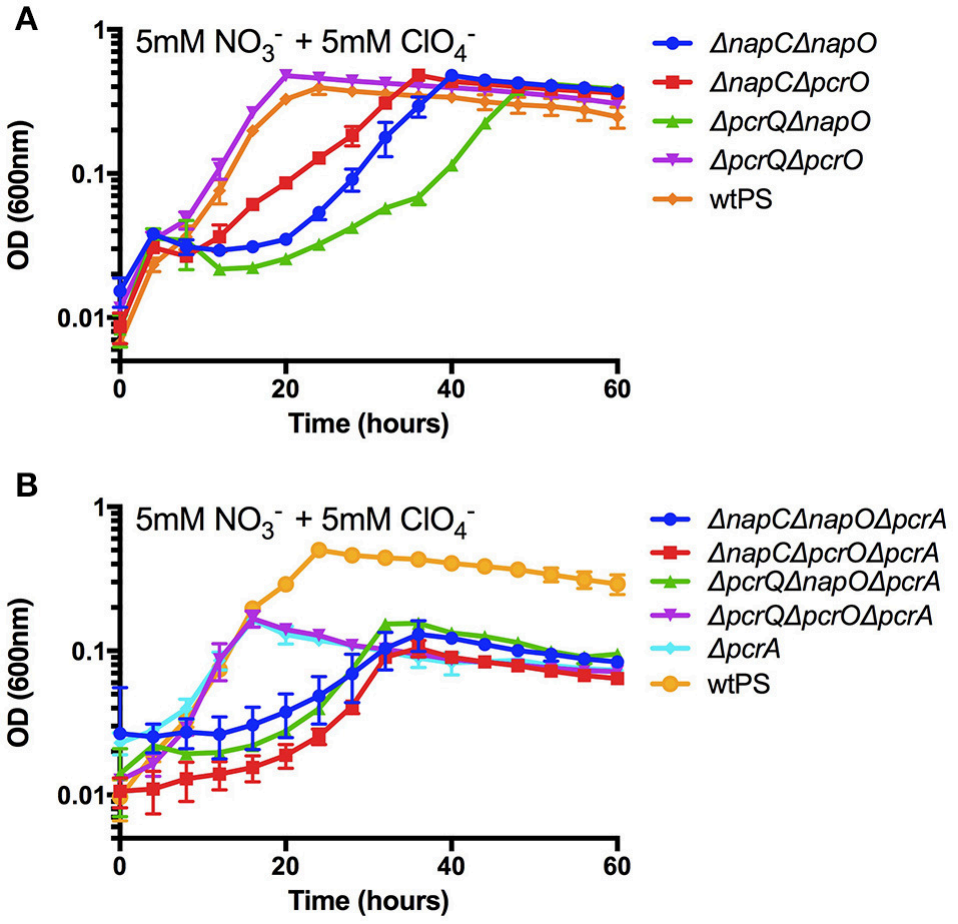
Growth of wtPS, Δ*napC*Δ*napO*, Δ*napC*Δ*pcrO*, Δ*pcrQ*Δ*napO*, and Δ*pcrQ*Δ*pcrO* **(A)**, and Δ*pcrA*, Δ*pcrA*Δ*napC*Δ*napO*, Δ*pcr*Δ*napC*Δ*pcrO*, Δ*pcrA*Δ*pcrQ*Δ*napO*, and Δ*pcrA*Δ*pcrQ*Δ*pcrO* **(B)** on LMM medium with 5 mM nitrate and 5 mM perchlorate. Error bars represent standard deviations of biological triplicate samples.

Based on our results, there are four viable electron transfer routes from the quinone pool to PcrABC and NapAB as exemplified in Figure S2. To show that all four routes are functional and can sustain growth, we constructed four double deletion mutants, *napC/napO, napC/pcrO, pcrQ/napO*, and *pcrQ/pcrO* (Δ*napC*Δ*napO*, Δ*napC*Δ*pcrO*, Δ*pcrQ*Δ*napO*, and Δ*pcrQ*Δ*pcrO*). These combinatorial double mutants in either the perchlorate or nitrate reduction pathways abolished all electron bifurcations and result in a single electron transfer route from the quinone pool to either PcrABC or NapAB (Figure S2). The growth phenotypes of these mutants were determined under nitrate and perchlorate diauxic conditions. The purpose of the diauxic condition was to ensure that the induction of both denitrification and perchlorate reduction pathways. Under the conditions tested, all four mutant strains were viable and able to grow to the same optical density as wtPS (Figure 4A). These results suggested that all four electron transfer routes are capable to transfer electrons to PcrABC.

To conclusively demonstrate Δ*napC*Δ*napO*, Δ*napC*Δ*pcrO*, Δ*pcrQ*Δ*napO*, and Δ*pcrQ*Δ*pcrO* can also transfer electrons to NapAB, four PS triple deletion mutants that contain *pcrA* deletion in the background of *napCnapO, napC*Δ*pcrO, pcrQ*Δ*napO*, and *pcrQpcrO* (Δ*pcrA*Δ*napC*Δ*napO*, Δ*pcr*Δ*napC*Δ*pcrO*, Δ*pcrA*Δ*pcrQ*Δ*napO*, and Δ*pcrA*Δ*pcrQ*Δ*pcrO*) were constructed and assayed for growth under nitrate and perchlorate diauxic conditions, with Δ*pcrA* and wtPS as controls. The resulting growth phenotypes are shown in Figure 4B. All mutant strains reached a final optical density that was similar to Δ*pcrA*. A summary of the growth rates of these strains can be found in Table S6. Of note, in mutants that harbor only one electron transfer route to PcrABC or NapAB, disruption of *nap* gene(s) always resulted in defective growth kinetics, indicating while NapC and NapO can fully replace the role of PcrQ and PcrO, PcrQ and PcrO can only partially rescue a growth defect as a result of lacking NapC and NapO.

The concluding redundancies that exist in these pathways are summarized in Figure 1. While PcrC from the perchlorate respiratory chain seems to lack a functional homolog in the nitrate respiratory chain, the same is also true for PcrB. Neither PcrC (tetraheme *c*-type cytochrome) nor PcrB (iron-sulfur cluster protein) are similar to NapB (diheme *c*-type cytochrome). However, PcrABC and NapAB could be considered as functional homologs as they both carries out the final step in terminal electron acceptor reduction.

Although extensive, such electron transfer branching and redundancy has been observed in other microorganisms. In *Shewanella oneidensis*, the Mtr metal respiratory system is highly modular and redundant, and multiple electron transfer routes exist to reduce several terminal electron acceptors, such as DMSO, iron oxide, and ferric citrate (Coursolle and Gralnick, 2010). A later study showed that *S. oneidensis* produces several interconnected functional electron transfer chains simultaneously (Sturm et al., 2015). It has been proposed that the modular electron transfer machinery enables the organism to quickly adapt to a variety of environmental electron acceptors, as well as offers a fitness benefit in redox-stratified environments (Sturm et al., 2015). Also, the redundancies in these electron transfer chains have implications for the past and future co-evolution of these respiratory metabolisms.

### Rewiring PS to alter the nitrate/perchlorate preferential usage

**Figure 5:**
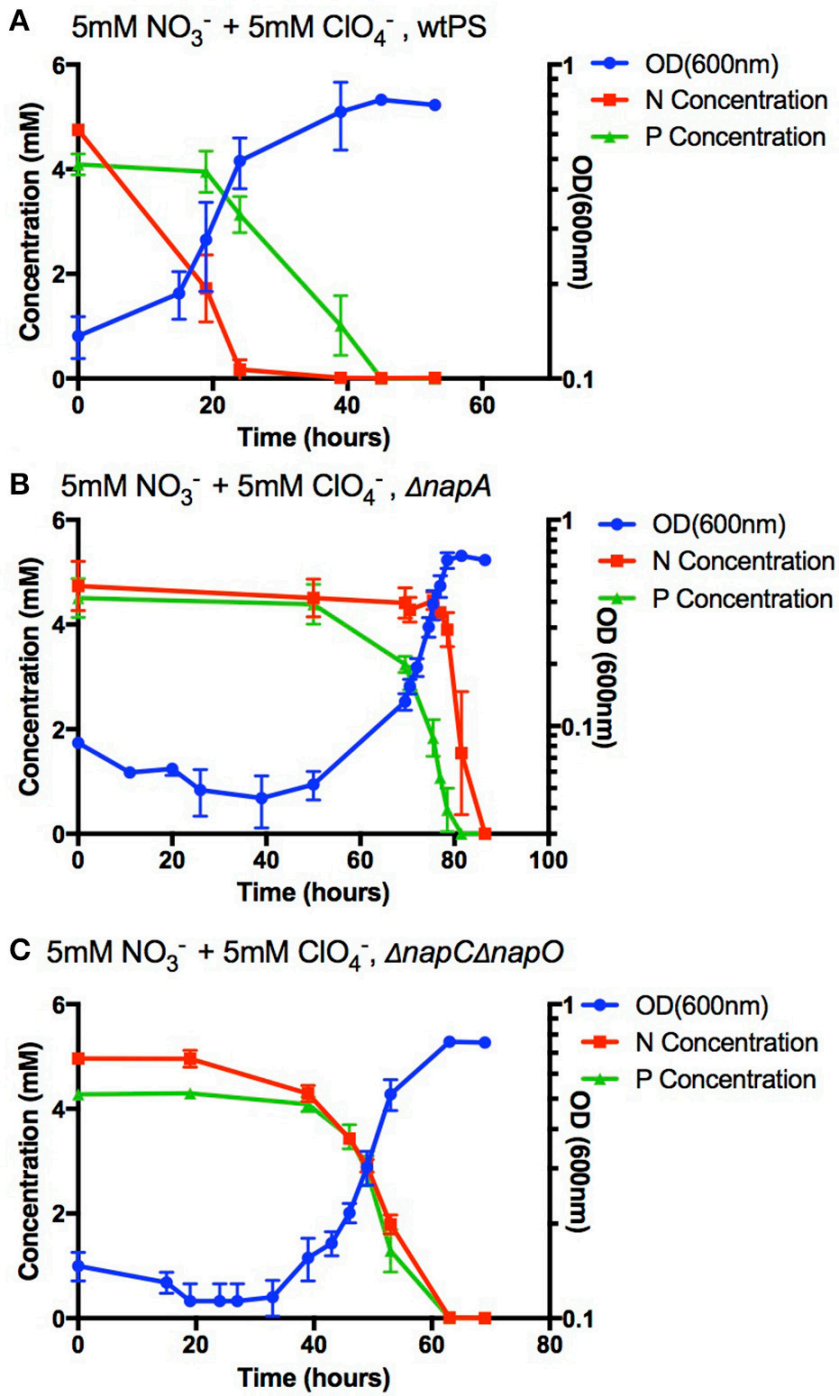
Growth and concentration of nitrate or perchlorate in cultures of wtPS **(A)**, Δ*napA* **(B)**, and Δ*napC*Δ*napO* **(C)**, in LMM medium with 5 mM nitrate and 5 mM perchlorate. Error bars represent standard deviations of biological triplicate samples.

The efficiency of perchlorate bioattenuation is often significantly impacted by the preferential use of nitrate by perchlorate reducing microorganisms (Coates and Achenbach, 2004). The rational construction of a perchlorate reducing strain that can preferentially reduce perchlorate or co-reduce both nitrate and perchlorate could significantly reduce operational costs and lead to more efficient remediation practices (Coates and Achenbach, 2004). We sought to leverage our understanding of electron flow in PS to alter the preferential usage of anaerobic electron acceptors. In a *napA* deletion strain (Δ*napA*), both nitrate and perchlorate reductions depend on the *in vivo* activity of perchlorate reductase PcrABC. Previous biochemical studies have demonstrated that a that PcrABC has a lower Km for perchlorate than for nitrate (Youngblut et al., 2016a). Consistent with this reasoning, during anaerobic growth with equimolar nitrate and perchlorate, Δ*napA* PS strains preferentially reduced perchlorate over nitrate (Figures 5A,B). Interestingly, a 60-h lag phase was observed in Δ*napA*, which is similar to the 20–40 h lag phase observed in wtPS when perchlorate was the sole acceptor (Figures 2B, 3). Despite the fact that under nitrate and perchlorate diauxic growth conditions wtPS always preferentially uses nitrate, the presence of nitrate actually enhances the onset of perchlorate reduction (see wtPS growth in Figures 2B,C, 3A, 5A; Chaudhuri et al., 2002; Melnyk et al., 2014). Presumably, this nitrate-dependent enhancement of perchlorate reduction is relying on the nitrate reduction pathway, which is interrupted in Δ*napA* (unlike Δ*napC* and Δ*napO*, expression of *pcr* proteins will not initiate nitrate reduction in Δ*napA* until depletion of perchlorate). In addition, the futile expression of a non-functional nitrate reduction complex in the absence of NapA, and the increased perchlorate concentration could also contribute to the prolonged lag phase. The rise of a suppressor mutant is implausible as the three biological replicates entered the log phase at a similar time, plus Δ*napA* would be expected to grow under nitrate and perchlorate diauxic conditions even in the absence of any additional mutation.

Another approach to altering electron acceptor preference is to maintain NapA expression, but disrupt expression of electron carriers to NapAB, namely NapC and NapO. In the double deletion mutant Δ*napC*Δ*napO*, nitrate reduction depends solely on the expression PcrQ and PcrO, which functionally replace NapC and NapO in the electron transport pathway. Thus, under diauxic growth conditions, reconstitution of functional nitrate reductase respiratory complexes comprised of NapAB-PcrQO and nitrate reduction will only occur upon expression and reconstitution of functional perchlorate reductase complexes comprised of PcrABCOQ. Consistent with this model, Δ*napC*Δ*napO* reduced both nitrate and perchlorate concomitantly (Figure 5C). It is important to note, that in this case, we do not know the extent to which nitrate may be co-reduced by PcrABCOQ in Δ*napC*Δ*napO* strains, but in Δ*napA* nitrate was only reduced by PcrABC after complete removal of perchlorate, suggesting that reduction of nitrate reduction by NapA is likely a major pathway (Figures 5B,C).

### Important outstanding questions related to regulation of nitrate and perchlorate reduction

While we have successfully identified redundant electron carriers in the nitrate and perchlorate respiratory chains in this work, independent regulation of nitrate, and perchlorate respiratory pathways plays an important role in controlling environmental electron acceptor preferential usage. As such, dissecting regulatory mechanisms remains an important goal for future studies. In nitrate reducing alpha- and gamma-proteobacteria, nitrate metabolism is globally regulated by the Crp/Fnr family of transcriptional regulators that sense redox changes (Lambden and Guest, 1976; Arai et al., 1997; Mesa et al., 2003; González et al., 2006), as well as the NarXL and NarQP two component systems that sense environmental activators, such as nitrate and nitrite (Rabin and Stewart, 1993; González et al., 2006). However, there is little known about the nitrate regulatory circuit in betaproteobacteria (e.g., PS). The nitrate/nitrite sensors NarXL and NarQP are absent in PS. Multiple Crp/Fnr transcriptional regulators are present in PS, but there is difficulty in predicting which transcriptional regulator is involved in nitrate metabolism regulation.

The perchlorate metabolism regulatory circuit is also not well-characterized in PS. The PAS domain containing the protein PcrP, histidine kinase sensor PcrS, and response regulator PcrR comprise the putative transcriptional regulatory system that senses internal and/or external signals (Melnyk et al., 2014). Based on homology to other systems, PcrS is likely regulated by PcrP to influence the phosphorylation state of PcrR which in turn interacts with RpoN to induce transcription of the *pcr* operon (Melnyk et al., 2014). However, the regulatory signal(s) that influence *pcr* operon induction via PcrPSR transcriptional regulatory system are still unknown.

## Conclusion

Nitrate often co-occurs in perchlorate-contaminated environments. As such, nitrate inhibition of perchlorate metabolism presents an obstacle in the bioattenuation of perchlorate in these environments, as electron donor amendment will stimulate unproductive nitrate reduction rather than perchlorate reduction. This study improves our understanding of nitrate and perchlorate respiratory pathways in DPRB and identifies key functionally redundant electron carrier proteins in these pathways. These redundancies are important to consider for the past and future co-evolution of perchlorate and nitrate respiratory pathways as well as other oxyanion respiratory pathways by related enzymes. We leverage the redundancy in PS to construct mutant strains with altered electron acceptor preference. While thorough knowledge of the regulatory signals that influence the expression of nitrate and perchlorate respiratory pathways remains important, this study demonstrates that nitrate/perchlorate electron acceptor preference can be altered by “rewiring” the electron transport chains without mutation of regulatory pathways.

## Author contributions

OW, RM, MM-K, MY, HC, and JC planned and designed the experiments. OW and RM performed the experiments and analyzed the data. OW drafted the manuscript. OW, HC, and JC finalized the manuscript.

### Conflict of interest statement

The authors declare that the research was conducted in the absence of any commercial or financial relationships that could be construed as a potential conflict of interest.

## Supplementary material

Figure S1 Growth of Δ*napG*Δ*napH* on LMM medium containing nitrate (N), perchlorate (P), both nitrate and perchlorate (PN).

Figure S2 A model illustrating four possible electron transfer routes to from quinone pool to PcrABC respiratory complexes in *Azospira suillum* PS. Arrows indicate electron transfer pathways. **(A–D)** Four proposed electron transfer pathways to PcrA.

Table S1 Primers used in this study; the names of all primers and sequences used in this study are listed here; a brief description of the primer is also supplied.

Table S2 Strains used in this study; all strains are listed here; a brief description of strain genotype is also supplied.

Table S3 Plasmids used in this study; plasmids are listed here by name, and the markers carried are described, along with a brief description and source.

Table S4 Specific growth rates (hr^−1^) of different mutants in Figure 2.

Table S5 Specific growth rates (hr^−1^) of different mutants in Figures 3A,B.

Table S6 Specific growth rates (hr^−1^) of different mutants in Figure 4.
