# Supplementary figures and images for: Functional Redundancy in Perchlorate and Nitrate Electron Transport Chains and Rewiring Respiratory Pathways to Alter Terminal Electron Acceptor Preference
Source: Front Microbiol. 2018 Mar 6;9:376. doi: 10.3389/fmicb.2018.00376 (PMC5845722; doi:10.3389/fmicb.2018.00376)

OD (600nm)

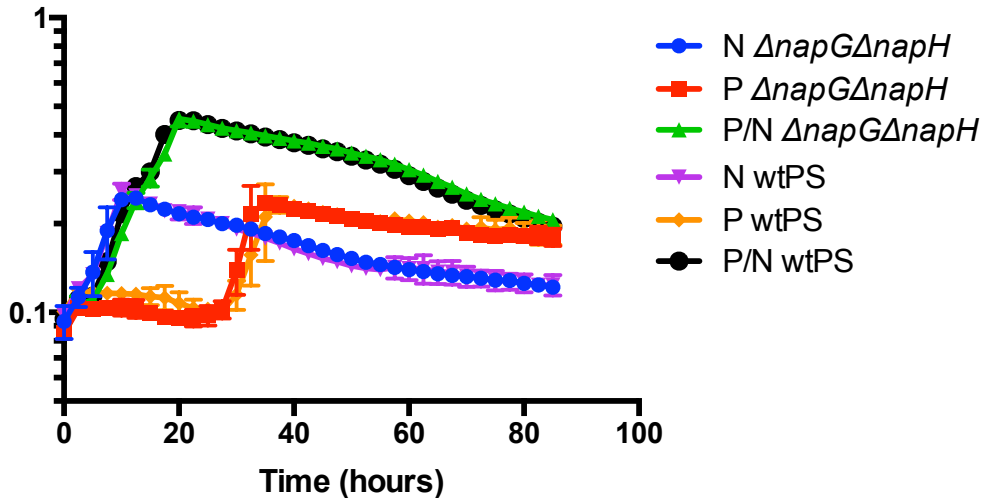

Supplement: Figure S1 — Growth of ΔnapGΔnapH on LMM medium containing nitrate (N), perchlorate (P), both nitrate and perchlorate (PN). [file Image1.PDF]

Four alternatives for  
electron transfer to PcrA

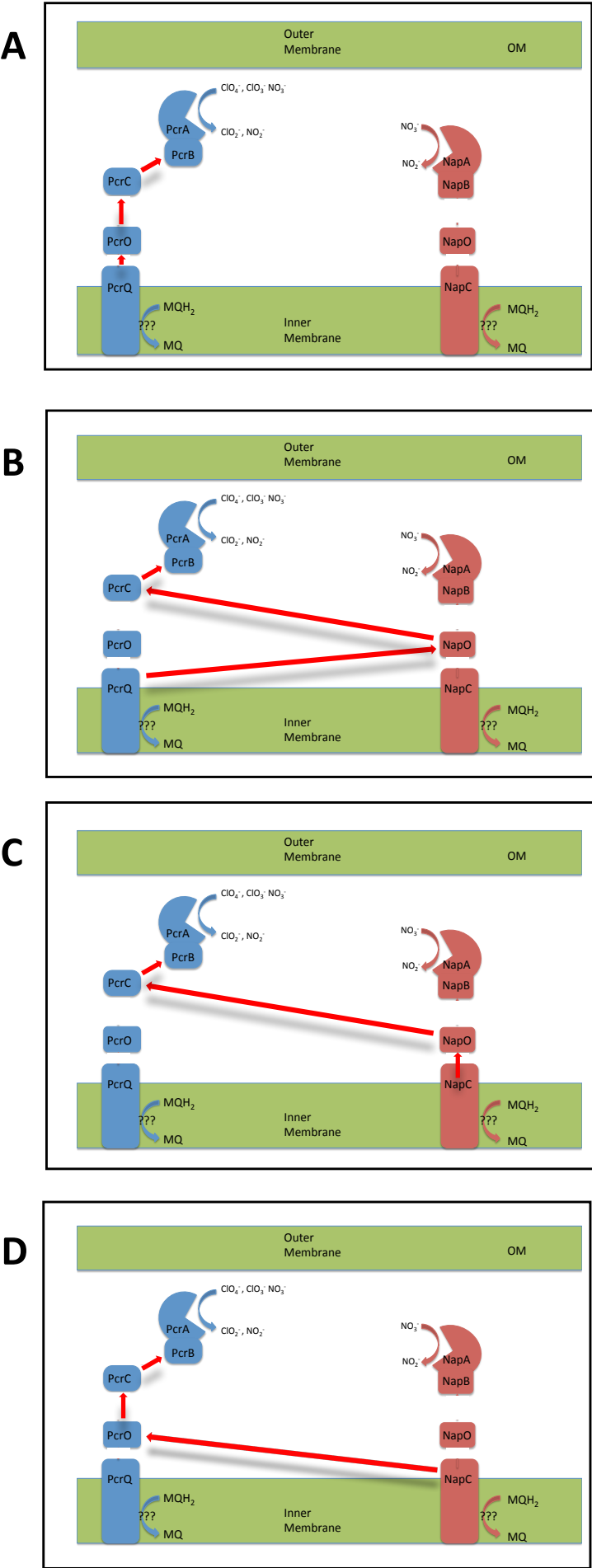

Supplement: Figure S2 — A model illustrating four possible electron transfer routes to from quinone pool to PcrABC respiratory complexes in Azospira suillum PS. Arrows indicate electron transfer pathways. (A–D) Four proposed electron transfer pathways to PcrA. [file Image2.PDF]
